# SOCIAL RESOURCES, COGNITION, AND MENTAL HEALTH DURING THE COVID-19 PANDEMIC

**DOI:** 10.1093/geroni/igac059.119

**Published:** 2022-12-20

**Authors:** Louise Hawkley, Laura Finch, Linda Waite, Ellen Compernolle

**Affiliations:** NORC at the University of Chicago, Chicago, Illinois, United States; NORC at the University of Chicago, Chicago, Illinois, United States; National Opinion Research Center (NORC at Chicago), Chicago, Illinois, United States; NORC at the University of Chicago, Chicago, Illinois, United States

## Abstract

This study assesses the extent to which changes in mental health among older adults from pre- to during the pandemic varied by cognitive functioning and the role that decreases in social resources played in this association. We use data from the National Social Life, Health, and Aging Project (NSHAP)—a population-based panel study of older U.S. adults that has surveyed respondents every 5 years since 2005—and the NSHAP COVID-19 supplement, conducted between September 2020 and January 2021 (N=2,672). Results from linear regression models suggest that (1) higher cognitive functioning in 2015 was associated with greater loneliness (β=-0.03; p<.05) during the pandemic; (2) this association is explained in part by a decrease in emotional support during COVID-19 (β=0.94; p<.001); and (3) cognitive status did not moderate links between social resources with happiness nor loneliness. Results emphasize the importance of social resources for older adults’ mental health, regardless of cognitive ability.

